# Physicochemical Changes of Apoferritin Protein during Biodegradation of Magnetic Metal Oxide Nanoparticles

**DOI:** 10.1021/acsami.4c12269

**Published:** 2024-09-17

**Authors:** Ehsan Rahimi, Amin Imani, Donghoon Kim, Mohammad Rahimi, Lorenzo Fedrizzi, Arjan Mol, Edouard Asselin, Salvador Pané, Maria Lekka

**Affiliations:** † Department of Materials Science and Engineering, Delft University of Technology, Mekelweg 2, 2628 CD Delft, The Netherlands; ‡ Department of Materials Engineering, The University of British Columbia, Vancouver, British Columbia V6T 1Z4, Canada; § Laboratory for Multiscale Materials Experiments, Paul Scherrer Institute, Forschungstrasse 111, Villigen 5232, Switzerland; ∥ Department of Mechanical Engineering, McMaster University, Hamilton, Ontario L8S 3L8, Canada; ⊥ Polytechnic Department of Engineering and Architecture, University of Udine, 33100 Udine, Italy; # Multi-Scale Robotics Lab, Institute of Robotics and Intelligent Systems, Department of Mechanical and Process Engineering, ETH Zurich, Tannenstrasse 3, Zurich 8092, Switzerland; ∇ CIDETEC, Basque Research and Technology Alliance (BRTA), Donostia-San Sebastián 20014, Spain

**Keywords:** apoferritin protein, biodegradation, oxide nanoparticles, protein surface potential, Kelvin probe force microscopy

## Abstract

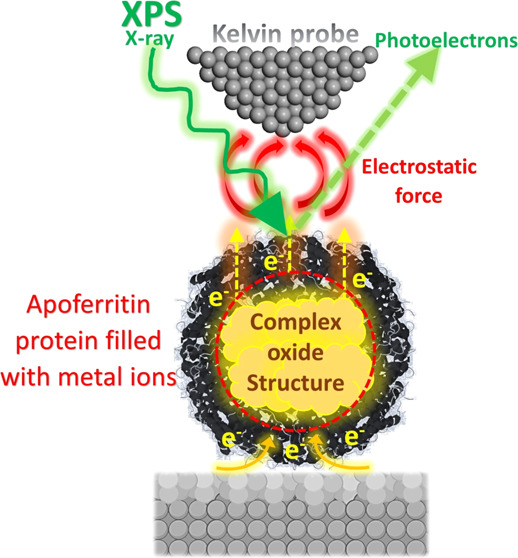

The biodegradation of therapeutic magnetic-oxide nanoparticles (MONPs) in the human body raises concerns about their lifespan, functionality, and health risks. Interactions between apoferritin proteins and MONPs in the spleen, liver, and inflammatory macrophages significantly accelerate nanoparticle degradation, releasing metal ions taken up by apoferritin. This can alter the protein’s biological structure and properties, potentially causing health hazards. This study examines changes in apoferritin’s shape, electrical surface potential (ESP), and protein-core composition after incubation with cobalt-ferrite (CoFe_2_O_4_) oxide nanoparticles. Using atomic force microscopy (AFM) and scanning Kelvin probe force microscopy (SKPFM), we observed changes in the topography and ESP distribution in apoferritin nanofilms over time. After 48 h, the characteristic apoferritin hole (∼1.35 nm) vanished, and the protein’s height increased from ∼3.5 to ∼7.5 nm due to hole filling. This resulted in a significant ESP increase on the filled-apoferritin surface, attributed to the formation of a heterogeneous chemical composition and crystal structure (γ-Fe_2_O_3_, Fe_3_O_4_, CoO, CoOOH, FeOOH, and Co_3_O_4_). These changes enhance electrostatic interactions and surface charge between the protein and the AFM tip. This approach aids in predicting and improving the MONP lifespan while reducing their toxicity and preventing apoferritin deformation and dysfunction.

## 1 Introduction

In recent decades, iron-based magnetic nanoparticles, particularly those doped with elements such as cobalt (Co),^1,2^ bismuth (Bi),^3,4^ manganese (Mn),^5,6^ nickel (Ni),^7^ calcium ion (Ca^2+^),^8^ and lanthanum ion (La^3+^)^9^ have garnered significant attention in the environmental and biomedical fields. These doped nanomaterials offer several advantages, including improved image monitoring, enhanced efficacy in contaminant degradation, notable catalytic or electrochemical reactions, and reduced mass for injections.^10,11^ Among the several iron-based magnetic nanoparticles, cobalt-ferrite oxide (CoFe_2_O_4_, CFO) nanoparticles have been extensively studied across all branches of engineering, medicine, and subdisciplines due to their magnetic properties (high curie temperature, large magnetocrystalline anisotropy constant K_1_, high coercivity H_c_, more saving power at high frequencies), chemical stability, mechanical hardness, and suitable catalytic properties.^12,13^ Particularly in medical applications, CFO nanoparticles have the ability to be utilized in the human body environment for transport, immobilization, and marking of biological species.^6^ Despite their high chemical stability and long-term durability, CFO oxide nanoparticles are vulnerable to degradation and metal ion release under various environmental and operational conditions.^14,15^

The human physiological environment is a complex medium composed of various ions, cells, macrophages (including splenic and hepatic macrophages), and a range of protein molecules, such as fibronectin, human serum albumin (HSA), apoferritin, and ferritin. Therefore, the physicochemical interaction of these biological species with CFO oxide nanoparticles gradually reduces their functionality and efficacy due to the biodegradation process and release of toxic Fe and Co ions.^14,16,17^ In the case of CFO oxide nanoparticles, Co ions released from CFO have the potential to induce the formation of reactive oxygen species (ROS), oxidative DNA damage, and oxidized proteins.^18^ In addition, the excess released Fe and Co ions can be stored for incorporation by proteins like apoferritin or ferritin and hemoglobin molecules.^19^ Cellular ferritin is an iron storage protein that can be found in all animals, plants, and bacteria; in the human body, it can be detected in the spleen, liver, inflammatory phases, and close to intercellular iron-based oxide nanoparticles.^17^ The main function of ferritin protein is the collection of excess iron ions in the human body in the form of ferrihydrite phosphate (FeOOH)_8_ (FeOPO_3_H_2_) in its core in order to prevent iron accumulation in human physiological media.^20^ The ferritin protein is made up of 24 subunits that enclose an aqueous cavity.^21^ This cavity can hold up to 4500 iron atoms in the form of an iron mineral, traditionally identified as ferrihydrite.^21^ Therefore, the ferritin molecules can play a substantial role in physicochemical interactions and magnetic efficiency of CFO nanoparticles, especially the Fe and Co ion releases and storing processes.

Many studies have been carried out on apoferritin and holoferritin protein adsorption or desorption on solid surfaces. These studies include aspects such as the proteins’ conformational rearrangement, electronic properties or electron-transfer mechanism(s),^22,23^ and the extent of various metal ion (Fe, Co, Ag, Au, Cu, Mn, Ni)^24−28^ uptake or release. A wide range of techniques are used in these investigations, including electrochemical measurements (cyclic voltammetry),^19^ ex situ^29^ and in situ^23^ atomic force microscopy (AFM), atomic force spectroscopy (AFS),^23^ current sensing-AFM (CS-AFM),^30^ magnetic force microscopy (MFM),^31^ scanning tunneling microscopy (STM),^25^ scanning tunneling spectroscopy (STS),^25^ X-ray photoelectron spectroscopy (XPS),^24^ quartz crystal microbalance measurements (QCMs),^32^ transmission electron microscopy (TEM),^27^ and simulation studies.^28^ Among the various scanning probe microscopy techniques (e.g., STM, AFM, AFS, and CS-AFM), scanning Kelvin probe force microscopy (SKPFM) is a distinctive approach because it has high surface sensitivity, especially in organic material subjects. The SKPFM technique with high lateral resolution (from μm^33^ to nm^34^ and Å^35^) can detect the electrical surface potential and/or surface charge distribution on single molecules in biological and chemical systems.^35−37^ This technique stands out for its minimal contact and charge injection or disturbance to the sample, which is a crucial parameter, especially in the study of soft organic and biological species.^33,38^ Depending on the protein charge distribution and polar residue structure and studied substrate, the electrical surface potential at the protein/matrix interface will be variable. In previous SKPFM studies on various biological systems, distinct values of surface potential differences were detected at the protein/matrix interface such as biotin+avidin/Au,^33^ Abl tyrosine kinase/Si,^34^ avidin/Si,^39^ bacteriorhodopsin/mica,^40^ and DNA-capped nanoparticles/Fe.^41^ Numerous investigations have focused on elucidating the biodegradation and remediation mechanisms of CFO oxide nanoparticles during their physicochemical evolution at the interfaces of protein nanobiofilms and oxide surfaces.^6,7,15,17,18^ However, the impact of the degradation and metal ion release from CFO oxide nanoparticles on the physicochemical evolution of apoferritin protein molecules on the nanoscale has been overlooked. Such changes can significantly influence the structure, stability, function, and interactions of these proteins.^21^

**Figure 1:**
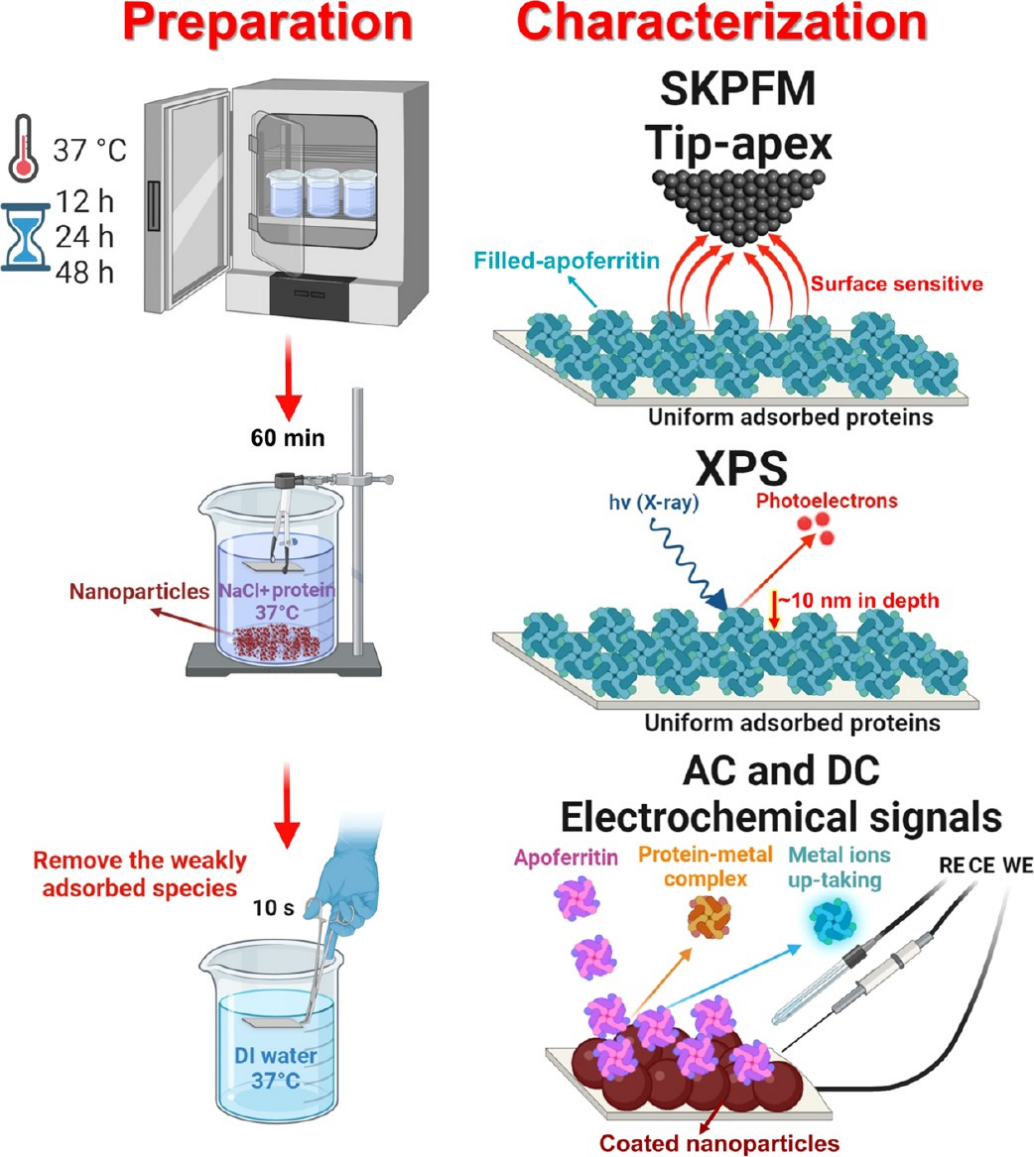
Schematic representation of the sample preparation procedure alongside the combined and systematic experimental approaches utilized in this research including AFM and SKPFM, XPS, and AC/DC multielectrochemical analyses.

This study aims to investigate the physicochemical interactions between apoferritin proteins and CFO oxide nanoparticles, focusing on the release and uptake of Fe and Co metal ions by apoferritin proteins over various incubation periods, including 12, 24, and 48 h. For achieving these purposes, combined atomic force microscopy (AFM), scanning Kelvin probe force microscopy (SKPFM), and X-ray photoelectron spectroscopy (XPS) were utilized to visualize the apoferritin molecular shape and its physicochemical evolutions overtime during the uptake of Fe and Co metal ions (Figure 1).

## 2 Experimental Procedure

### 2.1 Oxide Nanoparticle Fabrication and Sample Preparation

Cobalt-ferrite oxide (CFO) nanoparticles were synthesized according to our previous study.^15^ A conventional cobalt–chromium–molybdenum alloy (CoCrMo) was used as a substrate for the AFM/SKPFM analyses. It has an oxide layer consisting of mostly Cr_2_O_3_ and appropriate electrical conductivity for the SKPFM surface analysis of adsorbed protein monolayers.^42^ Additionally, a CoCrMo substrate was utilized since the other conventional substrates such as indium tin oxide (ITO),^19,43^ silica,^26^ mica,^29^ highly ordered pyrolytic graphite (HOPG), and pure gold^44^ cannot provide ideal conditions for the complete and perfect coverage of apoferritin protein molecules in the form of Langmuir isotherm adsorption, especially for protein immobilization.^42^ In addition, to avoid the mobilization of adsorbed protein molecules, most of these oxides or metallic surfaces require a surface functionalization process with organic compounds^23,45^ that are not considered in this work. A titanium alloy, Ti6Al4 V, known for its good biocompatibility and chemical stability, was utilized to ensure uniform adsorption and prevent protein mobilization during XPS analysis.^46^ After all of the substrate samples were polished to a mirror-like finish, they were washed with ethanol, ultrasonicated in acetone for 20 min, dried by air blowing, and eventually immersed in various apoferritin protein media.

### 2.2 Solution and Mixed Apoferritin/Oxide Medium Preparation

To evaluate the physicochemical interaction of CFO nanoparticles with apoferritin (apoferritin from equine spleen, with concentration 100 mg/mL, 0.2 μm filtered, Sigma-Aldrich) protein molecules, especially their metal ion uptake process, a mixed apoferritin + CFO solution was prepared: 500 μg/mL of apoferritin protein and 0.1 g/L of CFO nanoparticles were added to a 0.9% NaCl solution for different incubation times of 12, 24, and 48 h. The pH (detected by a pH meter, GLP 21 CRISON) and temperature of all CFO+apoferritin media were adjusted to 6 ± 0.1 and 37 ± 1 °C, respectively. In addition, a dark chamber was used for all of the incubations. The total release of Fe and Co metal ions over a 48 h incubation period was measured using inductively coupled plasma-optical emission spectrometry (ICP-OES, Agilent 5110), as shown in Figure S1.

### 2.3 Electron Microscopy Characterization

The microstructural and chemical composition of CFO nanoparticles were examined by scanning transmission electron microscopy (STEM, FEI Talos F200X) and energy-dispersive X-ray spectroscopy (EDXS), as shown in Figure S2.

### 2.4 Surface Topography and Electrical Potential by AFM and SKPFM

AFM and SKPFM surface analyses were utilized to visualize the morphological shape, surface potential, and/or surface charge (as a criterion for electronic properties of soft matter) of apoferritin proteins during physicochemical interactions with CFO nanoparticles with different incubation times including fresh apoferritin for 12, 24, and 48 h. All of these measurements were achieved on mirror-polished specimens with mean values of surface roughness 2.2 ± 1 nm (Figure S3). The immersion time of the CoCrMo alloy in the various incubated solutions was 60 min in order to obtain homogeneous adsorption of apoferritin proteins. The SPM device was a Nanoscope IIIa Multimode with an n-type-doped silicon pyramid single-crystal tip coated with PtIr5 (SCM-Pit probe, tip radius, and heights were 20 nm and 10–15 μm, respectively). The surface potential images were captured with a dual-scan mode: in the first scan, topography data were obtained using tapping mode, and in the second scan, the surface potential was detected by lifting the tip up to 60 nm. Topography and surface potential images were performed in the air atmosphere at 24 ± 1 °C with an approximate relative humidity (RH) of 28%, a pixel resolution of 512 × 512, a zero-bias voltage, and a scan frequency rate of 0.3 Hz. The histogram analysis based on the multimodal Gaussian distributions was used for a meaningful interpretation of the apoferritin protein morphology shape and the surface potential distribution after the various incubation times. All stages for the AFM/SKPFM sample preparation are schematically presented in Figure 1.

### 2.5 Chemical Surface Characterization by XPS

The XPS analyses were conducted with a Kratos AXIS Supra X-ray photoelectron spectrometer equipped with a monochromatic Al Kα source (15 mA, 15 kV). XPS probed the sample surface to a depth of 7–10 nm, with detection limits ranging from 0.1 to 0.5% atomic concentration depending on the element. The Kratos charge neutralizer system was applied to all of the specimens. Survey scan analyses were performed with an analysis area of 300 μm × 700 μm and a pass energy of 160 eV. High-resolution analyses used the same area but with a pass energy of 20 eV. Spectra were charge-corrected to the main line of the carbon 1s spectrum (adventitious carbon), set at 284.8 eV. Data were analyzed using CasaXPS software (version 2.3.26).

## 3 Results and Discussion

### 3.1 Energy Levels and SKPFM Surface Potential Signal of Unfilled and Filled Apoferritin

Using a low electron-transfer resistance probe in scanning Kelvin probe force microscopy (SKPFM) surface analysis, we can measure the local contact potential difference (ΔCPD) or work function energy (WFE) between a conductive AFM tip apex and the targeted sites on the substrate without any physical contact.^47^ According to the SKPFM principle, an appropriate external bias (*V*_DC_) must be applied to nullify the electrostatic force (*F*_EF_) during the electrical connection between the probe and the substrate, ensuring that the ΔCPD equals *V*_DC_. Typically, the *F*_EF_ between the AFM tip (during SKPFM analysis) and the sample surface is described as follows^47^
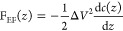
1where *z* is the direction normal to the surface of the studied sample, Δ*V* is *V*_DC_-ΔCPD, and d*c*/d*z* is the capacitance gradient between the tip apex and substrate surface. The electrical forces between the AFM tip and the studied surface in SKPFM measurements can be categorized into capacitance forces, arising from surface potential and dielectric screening, and Coulombic forces, resulting from static charges and multipoles.^48^ In a complex system, all of these factors directly affect the total ΔCPD and *V*_DC_ values detected by SKPFM.

**Figure 2:**
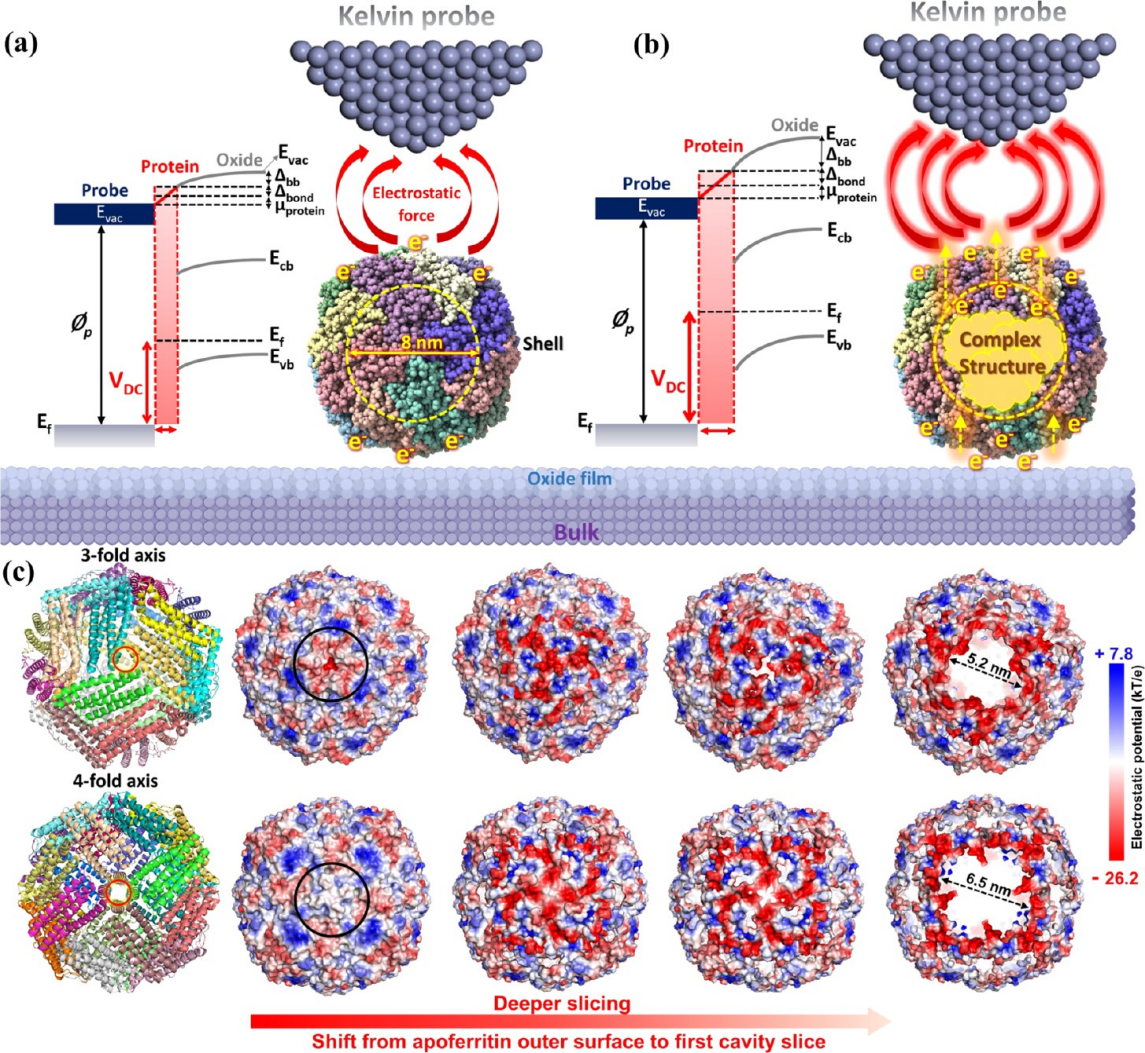
Schematic demonstration of the SKPFM principle along with the energy level diagram during the electrostatic interaction between a highly conductive AFM tip apex and (a) single fresh or (b) filled-apoferritin molecules on the oxide-bulk matrix at the atomic scale. The formation of a complex cluster in the internal cavity of the apoferritin molecule can significantly alter the energy level diagram, electrical surface potential, and intensity of electrostatic forces, and (c) the 3-fold and 4-fold axes of apoferritin molecules and their corresponding surface electrostatic potential in the different slices from the outset surface to closet or last slice to cavity (data generated by PyMoL and ChimeraX (PDB ID: 2W0O)) where positive, neutral, and negative amino acid residues are presented in blue, white, and red, respectively.

It is worth noticing that any physical or chemical evolutions on a solid surface such as surface microstructure and defects^46^ and adsorption or desorption of organic^49^ (especially soft matter^50^) or inorganic^51^ compounds/species strongly affect the local surface potential or WFE. Figure 2a demonstrates the fundamental principle of SKPFM surface analysis together with energy diagrams between an AFM tip apex and a single apoferritin molecule with relevant energy parameters including valence and conduction bands (*E*_vb_ and *E*_cb_), band-gap energy (*E*_g_), Fermi level (*E*_f_), and vacuum level (*E*_vac_).^52^ Following the adsorption and then the formation of a single, monolayer, or multilayer of biological species such as DNA and protein molecules on a solid matrix, the WFE can change to a new value due to electron transfer and structural relaxation at the interface.^53^ Therefore, in this study, a new arrangement of the energy levels will be established at the apoferritin biological molecule/oxide film that further influences the intensity of electrostatic forces and the magnitude of the capacitance (e.g., surface charge) owing to changes in local WFE or ΔCPD (Figure 2a).^49,54^ As shown in Figure 2a, the new electrostatic interaction and ΔCPD value between the AFM tip apex and adsorbed apoferritin protein are due to band bending (Δ_bb_, due to the formation of an accumulation region), interfacial bond (Δ_bond_, due to the new arrangement of electron density at the apoferritin/oxide film interface), and effective apoferritin molecular dipole (μ_protein_).^46^ Consequently, the new value of local surface potential on the adsorbed apoferritin protein–oxide complex (SP_protein–oxide_) can be defined as follows^49^
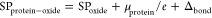
2

However, the metal ion uptake process by apoferritin toward its interior cavity (formation of a complex structure) causes an increase in the degree of the energy level misalignment (higher band bending) alongside changing the edge position of the energy levels.^25^ Subsequently, the intensity of the electrostatic forces and especially the ΔCPD value between the tip apex and the filled-apoferritin–oxide surface increases (Figure 2b). Comparing the higher ΔCPD for filled apoferritin vs unfilled in Figure 2, it is clear that the formation of the complex structure in the apoferritin cavity increases the charge transfer^52^ or conductivity^23^ at the protein/oxide film and results in a higher charge at the top surface of filled apoferritin. Figure 2c illustrates the electrostatic surface potential of the apoferritin molecule along the 3-fold and 4-fold axes, including various cross-sectional views from the outer surface to the innermost slice adjacent to the cavity. By gradually approaching the initial cavity slice part in both 3-fold and 4-fold axes, we can visualize a negative electrostatic potential distribution (approximately −26 *kT*/*e*) on diverse amino acid residues. This further triggers a strong electrostatic attraction between metal cations (e.g., Fe^2+^ and Co^2+^) and negative charge distribution on amino acids that facilitates the entrance of metal ions into the cavity and then the formation of complex compounds. The high affinity of apoferritin for the physicochemical interaction (protein–metal oxide complex and uptake process) is detectable using AC and DC electrochemical analyses in Figure S4. From the *E*–*I* curve, the addition of apoferritin increased the total current density (e.g., charge transfer) due to more metal–protein electrochemical interactions. This is further supported by the decrease in polarization resistance observed in the electrochemical impedance spectroscopy (EIS) curves in the presence of adsorbed apoferritin (Figure S4). Additionally, a remarkably high negative electrostatic potential can be seen on the outer surface of the 3-fold axis of apoferritin (marked by a black circle) that proves that the most preferential site for entering the metal ions into the apoferritin interior is the hydrophilic 3-fold channel.^55^

### 3.2 Morphology and Surface Potential of Apoferritin at Various Incubation Times

**Figure 3:**
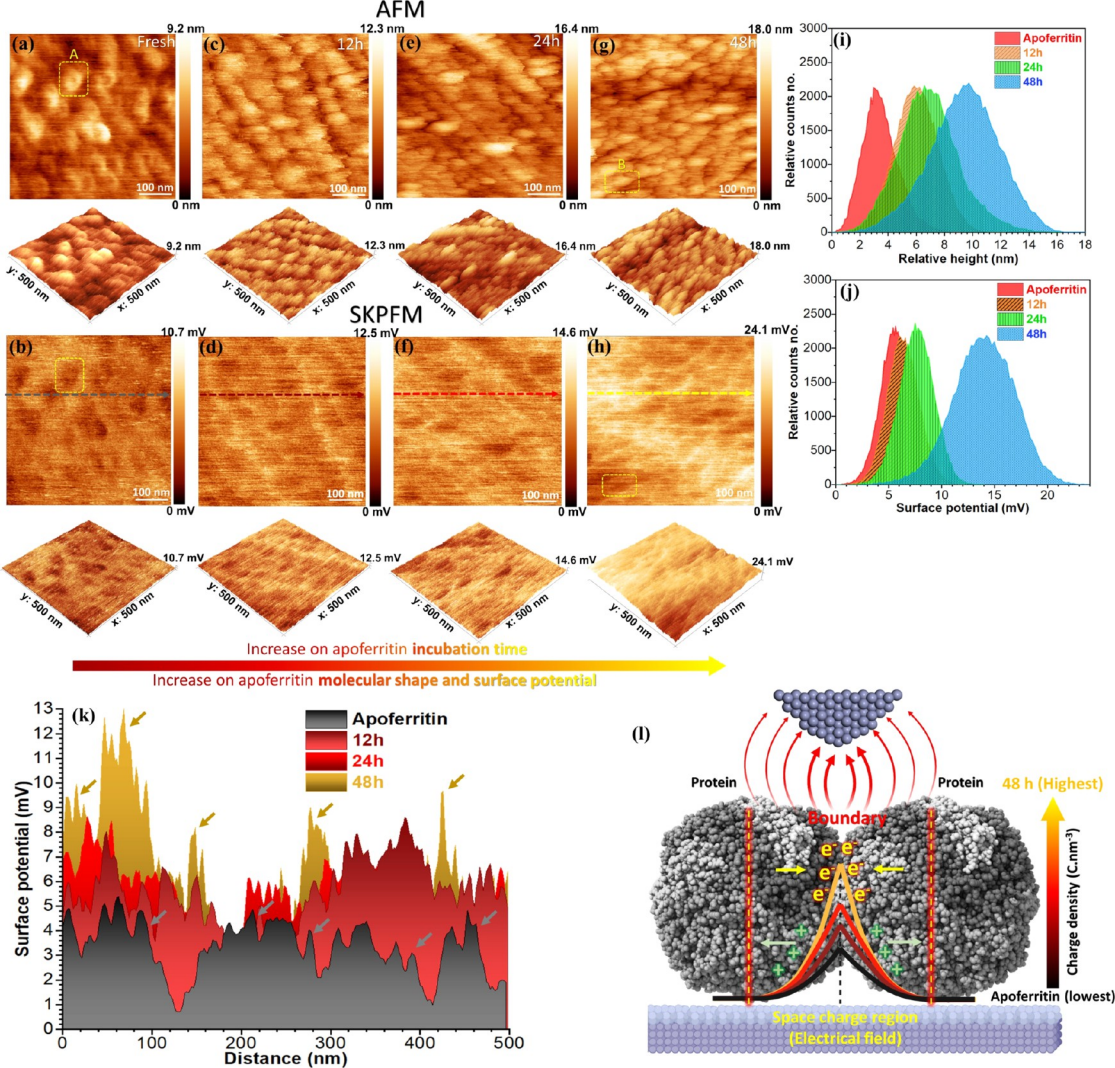
2D and 3D AFM (first row) and SKPFM (second row) images of the adsorbed apoferritin protein on oxide matrix in the different incubation times interacted by CFO nanoparticles including (a, b) fresh apoferritin or 0 times, (c, d) 12 h, (e,f) 24 h, and (g, h) 48 h; the environment was NaCl physiological solutions + CFO nanoparticles + apoferritin proteins at 37 °C, pH 6 ± 0.1, (i) relative height and (j) surface potential histograms that are related to AFM/SKPFM images, (k) the surface potential line profiles of boundaries at the protein/protein interface in different incubation times are obtained from panels (b, d, f, and h), (l) schematic representation of the energy level and charge accumulation at the boundary between two protein molecules in the different incubation times that directly impact the interfacial bonds and electrostatic force between the protein target site and AFM tip apex; note: the yellow rectangle containing selections A and B is designated for individual protein investigation in Figure 6.

Utilizing AFM and SKPFM, the adsorption morphology (including height (apoferritin diameter) and shape) and electrical surface potential distribution of the adsorbed-filled-apoferritin nanofilm on the oxide surface were revealed after various incubation times in the NaCl solution containing CFO nanoparticles, as shown in Figure 3a–h. A simple static immersion of the solid substrate in various apoferritin-incubated solutions demonstrated a fully uniform covering by apoferritin proteins that further indicates that apoferritin shows a high tendency to fill the available unoccupied surface sites.^56,57^ Hence, all adsorbed apoferritin surfaces are governed by the Langmuir isotherm adsorption mechanism.^46^ The topography image in Figure 3a evidently demonstrates an approximate homogeneous formation of apoferritin protein nanofilm with a slightly circular shape and a fairly dark hole appearing in the center of the apoferritin molecules (vide infra). Considering the corresponding electrical surface potential map in Figure 3b, there is a lower surface potential and/or surface charge distribution on the apoferritin nanofilm (dark quasi-circle regions) than on the conductive substrate (bright matrix).^34^ For different incubation times up to 48 h, the topography and shape of the adsorbed-filled apoferritin also gradually increased. For the apoferritin proteins incubated in a simulated medium containing aggressive Cl^–^ ions^58^ and CFO nanoparticles, particularly due to Fe and Co ion uptake by apoferritin,^45^ a newly modified protein topography and shape can be observed^31^ (Figure 3c,e,g).

Based on the histogram analysis of the topography and electrical surface potential maps presented in Figure 3i,j and calculated results in Figure S5, an increase in mean values and standard deviations of both topography and surface potential maps of adsorbed-filled apoferritin was detected with longer incubation times. This is seen by the shifting of histogram peaks of both topography and electrical surface potential to higher values (Figure 3i,j). Additionally, further AFM statistical analysis reveals an increase in surface roughness variation (*S*_a_) over incubation time, with values of 1.1 nm for fresh apoferritin (0 h), 1.4 nm after 12 h, 1.7 nm after 24 h, and 2 nm after 48 h. In the biological systems, it is known that the adsorbed single or nanofilm of protein^58,59^ or DNA^33,39^ molecules on the solid substrate (e.g., metals, oxides, conductive soft matter) demonstrate a lower electrical surface potential or surface charge distribution than the solid matrix. By prolonging the incubation time, the amount of filled apoferritin is increased, further affecting surface covering (increasing the mean value of the relative height distribution). Indeed, the apoferritin Co and Fe ion uptake affect the electronic and chemical properties of the apoferritin exterior shell.^29,60^ These well-known events^26,31^ are seen by our electrical surface potential and/or surface charge measurements at various incubation times,^39^ and they directly correlate to the evolution of these physical and chemical properties.

The electrical surface potential maps in Figure 3d,f,h further demonstrate an enhancement in the electrical surface potential and/or surface charge of all adsorbed apoferritin proteins. Specifically, a gradually increasing surface potential and/or charge becomes noticeable at boundaries between adsorbed proteins and the matrix (brighter with increasing incubation time). This effect becomes more pronounced when examining the random line profiles of surface potential differences across the horizontal direction at multiple protein/protein interfaces (indicated by arrows), as illustrated in Figure 3k. The surface potential difference values increased with incubation time, increasing from approximately 4 mV at 0 h (fresh apoferritin) to about 12 mV after 48 h. A higher surface potential difference (e.g.,*V*_DC_ or SP_protein–oxide_ at eq 2) will be established by extending the apoferritin incubation time due to the high interfacial bond (Δ_bond_) and effective protein molecular dipole (μ_protein_) that triggers the charge accumulation at the filled apoferritin/oxide interface. This new high interfacial bond or electronic interaction can affect the energy diagram level at the protein/oxide interface (higher band bending in Figure 2b),^61^ resulting in the new electrostatic dipoles, as detected at protein boundaries in filled-apoferritin conditions (Figure 3k,l).

### 3.3 Chemical Composition of Nonfilled and Filled-Apoferritin Nanofilm by XPS

**Figure 4:**
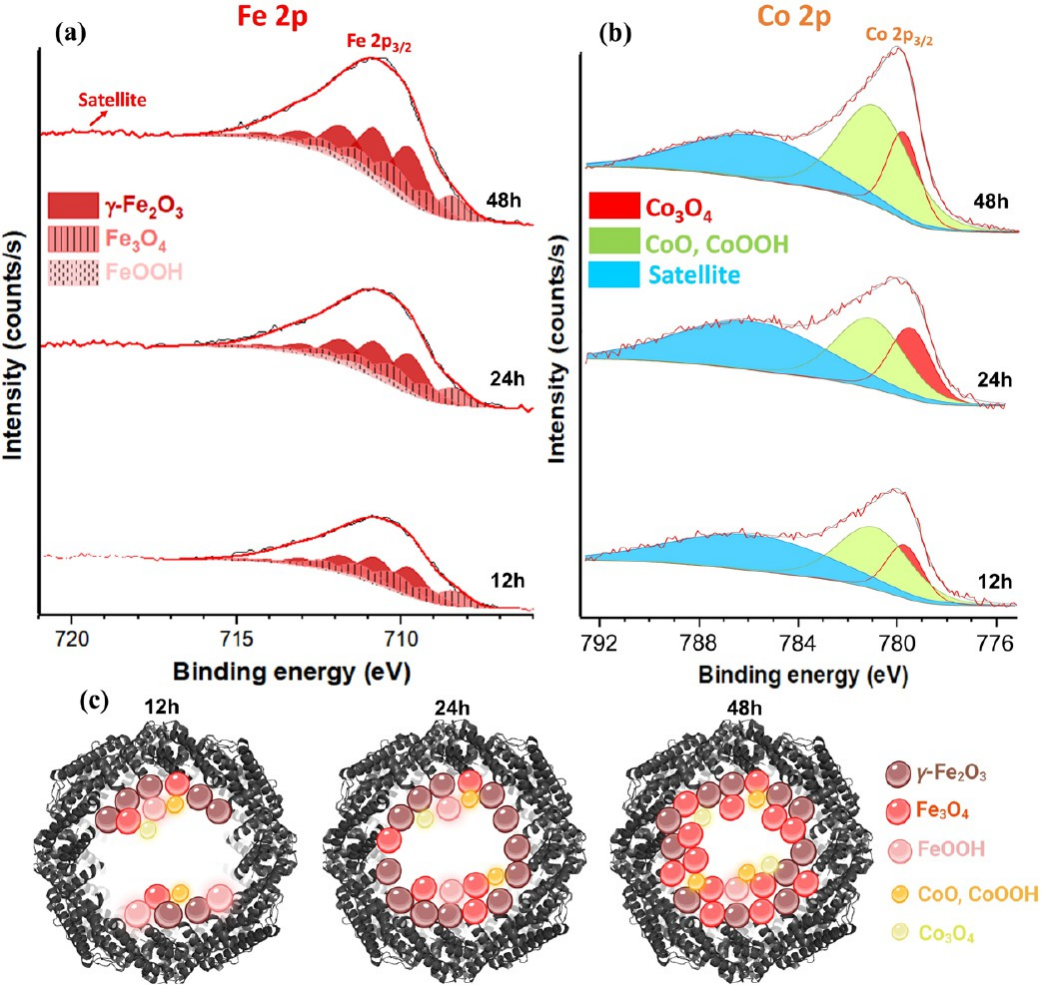
XPS spectra of (a) Fe 2p and (b) Co 2p electron energy regions from the core of the adsorbed-filled-apoferritin monolayers were detected after different incubation times. (C) A schematic representation of Fe and Co ion uptake by apoferritin during the different incubation times.

The elemental composition of adsorbed fresh apoferritin (nonfilled) alongside filled-apoferritin monolayers in various incubation times was characterized by XPS with a depth of ∼10 nm. The XPS signal of individual high-resolution spectra of Fe 2p and Co 2p specifically related to incubated apoferritin is presented in Figure 4a,b. No substantial signals of either Fe 2p and Co 2p were detected for adsorbed fresh apoferritin (nonfilled) in binding energy ranges of 700–1000 eV in the survey spectrum (Figure S6). However, significant multisignals related to both Fe 2p and Co 2p in the same binding energy range are apparent for adsorbed-filled apoferritin for the 12 h incubation time (Figure S6). The Fe 2p spectra collected from all filled-apoferritin monolayer samples were fit, with multipeaks revealing the multiplet splitting of the Fe^2+^ and Fe^3+^ cations.^62^ The Fe 2p_3/2_ peak for both Fe^2+^ and Fe^3+^ high-spin compounds has been accurately fitted using the Gupta and Sen multiplet structure,^67^ which accounts for spin–orbit and electrostatic interactions.

**Figure 5:**
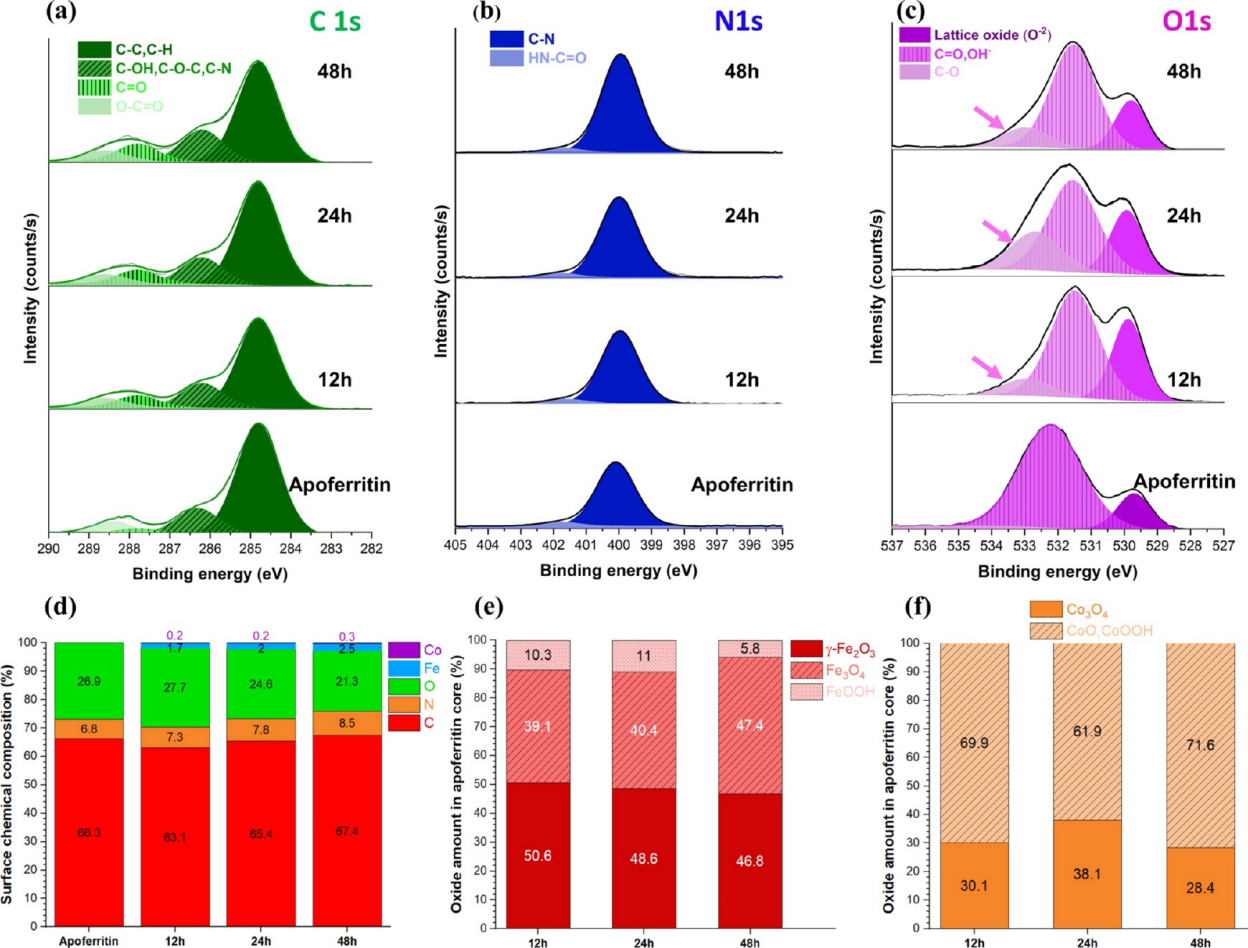
(a–c) XPS spectra of C 1s, N 1s, and O 1s electron energy regions from adsorbed apoferritin (nonfilled) and filled-apoferritin protein with different incubation times, (d) surface chemical composition of adsorbed apoferritin and filled-apoferritin protein monolayers with different incubation times, and amount of (e) iron and (f) cobalt oxide states in the apoferritin core during the various incubation times.

The binding energies of all fitted peaks are obtained in Table S1. Overall, these multideconvoluted peaks are grouped into three main iron oxides, irrespective of the incubation time, including hematite (γ-Fe_2_O_3_), magnetite (Fe_3_O_4_), and iron oxyhydroxide (FeOOH). Notably, within the ferritin protein, the oxidation of Fe^2+^ to Fe^3+^ initiates the formation of iron oxide due to the insolubility of Fe^3+^.^63^^63^ By increasing the incubation time, the total amount of Fe (Fe signal from the apoferritin core) increased from 1.7 atom % (12 h) to 2 atom % (24 h) and finally 2.5 atom % (48 h). This increase is accompanied by more formation of Fe_3_O_4_ and less formation of γ-Fe_2_O_3_ and especially FeOOH (Figures 4a and 5e) due to more reduction of FeOOH to Fe_3_O_4._^21,64,65^

As reported in earlier findings, the ferrihydrite phase in the ferritin core is more unstable to chemical reduction and mobilization than the magnetite phase.^21^ The XPS analysis from other studies indicated that the cluster core of filled apoferritin (AfFtnAA, Fe4800) consists of 62% Fe^3+^ and 38% Fe^2+.^^66^

There is a very low amount of Co (0.2–0.3 atom %) in all adsorbed-filled-apoferritin monolayers, which further confirms the lower tendency of the apoferritin molecule for uptake and oxidation of Co^2+^. Nevertheless, the Co 2p spectra exhibit two distinct peaks at 779.54 and 780.72 eV, corresponding to Co_3_O_4_ and either CoO or CoOOH.^67^ Additionally, all binding energies related to various incubation times are presented in Table S2. This aligns with the prior research utilizing STEM/XRD, which detected the presence of mixed cobalt oxides, including Co_3_O_4_ and CoOOH, with plane directions (220) and (311) for Co_3_O_4_ and (101) and (012) for CoOOH, respectively. These peaks maintain a relatively constant intensity in 12 h and 24 h incubation periods (Figures 4b and 5f). However, the intensity of both peaks enhanced at a 48 h incubation time. Hence, the surface of apoferritin protein provides high charge density regions that act as nucleation sites (Section 3.1), facilitating mineralization through complementary electrostatic interactions between the protein and the forming mineral nucleus^68^ (Figure 4c).

The C 1s, N 1s, and O 1s spectral signals in Figure 5a–d indicate that increasing the incubation time results in greater adsorption of filled apoferritin, as seen by the increase in C and especially N content. This observation aligns with the topography histogram shown in Figure 3i. Additionally, the decrease in the oxygen content with increasing incubation times suggests a more extensive coverage of the matrix, leading to a reduced substrate oxygen signal. The C 1s spectra consist of four peaks including C–C and C–H at 284.8 eV; C–OH, C–O–C, and C–N^64^ at 286.20 eV; C=O at 287.80 eV; and O–C=O 288.60 eV. By increasing the incubation time, all signals at 286.20, 287.80, and 288.60 eV are enhanced (Figures 5a and S7). This enhancement is attributed to greater apoferritin adsorption, increased uptake of Fe ions, and their subsequent oxidation, which result in a higher content of C=O and O–C=O bonds within the apoferritin molecules. The N 1s spectra of all adsorbed apoferritin monolayers indicate two individual peaks at 399.9 and 401.7 eV assigned to the C–N and NH_4_^+^ (it may be NH–C=O^64^), respectively. The O 1s spectra of filled-apoferritin monolayers at 12, 24, and 48 h indicate the emergence of a new peak at a binding energy of 532.94 eV (C–O peak in Figure 5c). This peak intensifies with longer incubation times (Figure S7), indicating increased interaction between the amine groups and iron hydroxide, mainly oxides (higher formation of the Fe_3_O_4_ phase).^21^

### 3.4 Nanometric Approach to Apoferritin Molecular Shape and Surface Potential Evolutions

**Figure 6:**
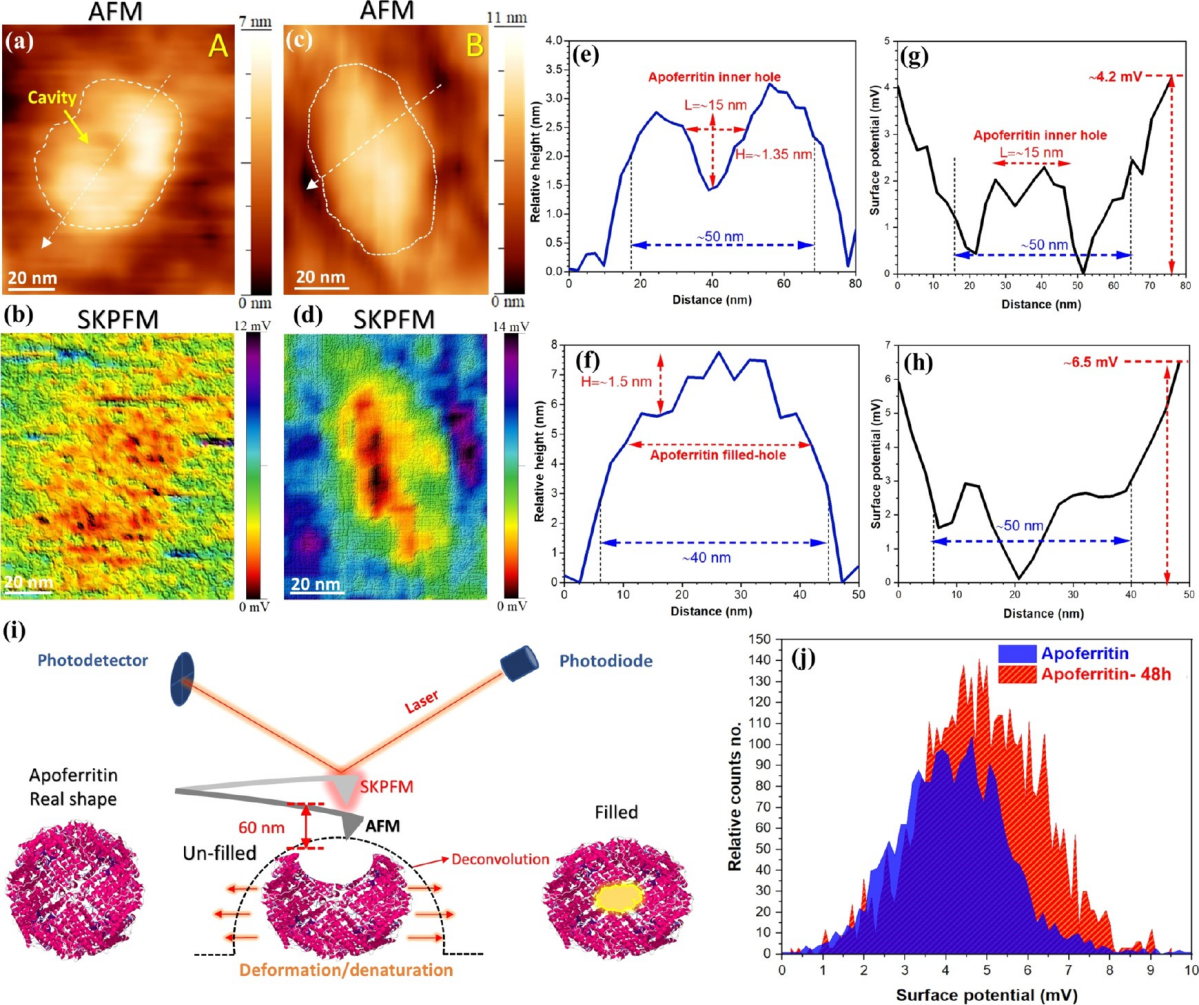
High-resolution AFM and SKPFM maps of (a, b) single fresh apoferritin and (c, d) 48 h incubated apoferritin from Figure 3 (yellow marked rectangle), (e, f) topography, and (g, h) surface potential line profiles of a single apoferritin molecule extracted from AFM/SKPFM maps, (i) schematic illustration of the tapping mode of the AFM probe that significantly alters the real molecular shape of the apoferritin molecule due to AFM tip-compressive force and AFM tip broadening effect, and (j) electrical surface potential histograms that are related to SKPFM images in panels (b, d).

Figure 6 presents high-resolution SKPFM/AFM results. According to the AFM image of single fresh and 48 h incubated apoferritin and their corresponding line profiles in Figure 6e,f, both adsorbed single apoferritins exhibit an ellipsoidal shape with approximate identical length values of ∼40–50 nm. This apparent longer dimension of both fresh and 48 h incubated apoferritin molecules with respect to standard apoferritin dimensions (external and internal diameters 12 and 8 nm (internal empty cavity), respectively) is related to the AFM tip–sample convolution, especially deformation due to the AFM tapping mode, as presented in the schematic image of Figure 6i. Due to this fact, the internal empty cavity of the fresh apoferritin molecule (yellow arrow in Figure 6a) was discerned with length and relative height values of ∼15 and ∼1.35 nm, respectively (Figure 6e). However, this internal cavity is not detectable in apoferritin incubated for 48 h. Only an increase in diameter, close to 1.5 nm in the central part, was observed (Figure 6f). Thereby, the total diameter value of a single fresh apoferritin molecule enhanced from ∼3.5 to ∼7.5 nm after 48 h of incubation. These detected values are precisely consistent with previous AFM studies that demonstrated that partly hollow ferritin (approximately apoferritin protein) molecules had diameter values of 3 ± 1 nm on a gold surface and the ferritin (filled-apoferritin molecules) molecules identified with apparent diameter and height values of 50 nm and 6–10 nm, respectively.^44^ They noticed that this considerably larger dimension is due to the denaturation of protein and the AFM tip-compressible interaction. Hence, the Fe and Co ion uptake by apoferritin fills its internal cavity (formation of Fe and Co oxides) and then increases its diameter value.

Based on the electrical surface potential map of a fresh apoferritin molecule shown in Figure 6b, a denatured structure can be observed. This denatured structure has a heterogeneous distribution and exhibits a lower surface potential or charge compared to the oxide layer. Normally, the electrical surface potential of biological molecules is strongly associated with the charge distribution and polar residue structure, particularly the pH and isoelectric point (IEP).^69^ Subsequently, a protein molecule may reveal an overall, negative, positive, or neutral charge depending on the ionization state of the main amino acid groups of the protein.^54^ The IEP value of apoferritin based on prior experimental analyses is around 4–4.8.^31,70^ Hence, the apoferritin molecule chemisorbed on the oxide layer decreased the electrical surface potential due to the presence of new potential steps and band bending at all energy levels (Section 3.1, e.g., *E*_vb_, *E*_cb_, *E*_f_, etc.). From the electrical surface potential map in Figure 6b, it is evident that the slightly unfolded apoferritin induced subunit dissociation, resulting in the formation of small nanometer-sized structures with varying surface potentials and charge distributions.^44^ This is consistent with a previous study that indicated that the gold nanoroughness, especially step edges, strongly influenced the morphology of adsorbed ferritin and its structure or chain formation.^44^

However, the adsorption morphology and electrical surface potential of apoferritin incubated for 48 h, as shown in Figure 6d, exhibit a more pronounced ellipsoidal shape compared to the fresh apoferritin depicted in Figure 6b. The histogram analysis of electrical surface potential in Figure 6j indicates the overall electrical surface potential differences (between the protein and substrate) in 48 h incubated apoferritin increased by shifting the histogram peaks to a higher value (from the mean value of ∼4–5 mV). Furthermore, the surface potential line profiles of single fresh apoferritin and filled apoferritin in Figure 6g,h disclose different values of 4.2 and 6.5 mV, respectively. Due to the formation of complex Fe and Co oxides within the internal cavity of apoferritin protein molecules (as indicated by the XPS results in Section 3.3), various semiconductor characteristics emerge. This results in enhanced charge transfer or electrical conductivity (as detected by SKPFM), owing to the reduced band-gap energy.^25^ Consequently, the complex metal core region facilitates increased conductivity to the protein polypeptide exterior shell, and vice versa. This interaction leads to a higher band bending within the oxide matrix, thereby increasing the surface potential. Ultimately, this results in stronger electrostatic forces between the AFM tip apex and the protein–oxide surface (Figure 2b).

## 4 Conclusions

In summary, at the nanoscale, we investigated the apoferritin molecular structure, electrical surface potential/charge, and core–shell chemical composition evolutions through the physicochemical interaction with magnetic-oxide nanoparticles during various incubation times. AFM/SKPFM analysis revealed a substantial enhancement in the topography and surface potential distribution of the adsorbed apoferritin nanofilm with increasing incubation times of 12, 24, and 48 h. These changes are due to the formation of complex Fe and Co oxides in the apoferritin internal cavity. Corresponding nanometric surface chemical composition measured by XPS showed an increase in the percentages of Fe (from 1.7 to 2 and 2.5%), Co (0.2 to 0.3%), and N (6.8 to 8.5%) over the same time intervals. These complex oxides might primarily be γ-Fe_2_O_3_, Fe_3_O_4_, CoO, CoOOH, and, to a lesser extent, FeOOH and Co_3_O_4_, particularly at 48 h of incubation. The formation of complex Fe and cobalt oxides in the internal cavity of apoferritin triggered higher band bending in the oxide matrix, increasing the electrical surface potential/charge, resulting in higher electrostatic forces between the AFM tip apex and adsorbed-filled apoferritin. These integrated experimental approaches can provide a detailed knowledge of how functionalized oxide nanoparticles affect apoferritin’s native structure and function. Moreover, this comprehensive approach helps predict the nanoparticles’ lifespan and toxicity, ensuring their safe and effective utilization in nanomedicine. Such research is crucial for advancing the safety and efficacy of nanotherapeutics, thereby enhancing clinical outcomes in nanomedicine applications.

## Supporting Information Available

ICP-OES analysis to quantify Fe and Co ion release after 48 h of incubation; TEM imaging and EDS elemental analysis of CFO nanoparticles; AFM topography imaging of the CoCrMo mirror-like surface; multielectrochemical analysis of the deposited CFO nanoparticle in apoferritin protein media; AFM and SKPFM histogram data, including mean values and standard deviations, and XPS survey spectra comparing fresh (nonfilled) and filled apoferritin after 12 h of incubation; binding energy of Fe 2p and Co 2p in adsorbed-filled apoferritin; and XPS analysis of C and O contents in adsorbed apoferritin monolayers at various incubation times (PDF)
