# Supplementary material for: Physicochemical Changes of Apoferritin Protein during Biodegradation of Magnetic Metal Oxide Nanoparticles
Source: ACS Appl Mater Interfaces. 2024 Sep 17;16(39):53299–310. doi: 10.1021/acsami.4c12269 (PMC11450718; doi:10.1021/acsami.4c12269)
Supplement: Supplementary file 1 — am4c12269_si_001.pdf [file am4c12269_si_001.pdf]

Supporting Information

Physicochemical Changes of Apoferritin Protein during Biodegradation of Magnetic Metal Oxide Nanoparticles

Ehsan Rahimi[a*] Amin Imani[b], Donghoon Kim[c], Mohammad Rahimi[d], Lorenzo Fedrizzi[e], Arjan Mol[a], Edouard Asselin[b], Salvador Pané[f], Maria Lekka[g]

[a]Delft University of Technology, Department of Materials Science and Engineering, Mekelweg 2, 2628 CD Delft, The Netherlands

[b]Department of Materials Engineering, The University of British Columbia, Vancouver, BC, V6T 1Z4, Canada

[c]Laboratory for Multiscale Materials Experiments, Paul Scherrer Institute, Forschungstrasse 111, Villigen, 5232 Switzerland

[d]Department of Mechanical Engineering, McMaster University, Hamilton, Ontario L8S 3L8, Canada

[e]Polytechnic Department of Engineering and Architecture, University of Udine, 33100 Udine, Italy

[f] ETH Zurich, Multi-Scale Robotics Lab, Institute of Robotics and Intelligent Systems, Department of Mechanical and Process Engineering ,Tannenstrasse 3, Zurich, 8092 Switzerland

[g]CIDETEC, Basque Research and Technology Alliance (BRTA), Donostia-San Sebastián, 20014 Spain

E.Rahimi: **e.rahimi-2@tudelft.nl**

Detecting the metal ion releasing by ICP-OES analysis

The inductively coupled plasma-optical emission spectrometry (ICP-OES, Agilent 5110) analysis was utilized to determine the concentration of released Co and Fe ions in an incubated solution containing nanoparticles (0.1g/L) and apoferritin proteins (500 µg/mL) for 48h. The incubated environment was kept at pH 6±0.1 and 37 °C in a dark chamber. The ICP-OES solution was prepared by precisely removing the remaining nanoparticles from the 48h incubated solution. Then the cleaned solution was properly prepared by acid digestion process before starting the analysis. The digestion process was performed on a hot plate in a clean baker by adding 2mL HNO_3 and 2mL H_2O_2 [1].

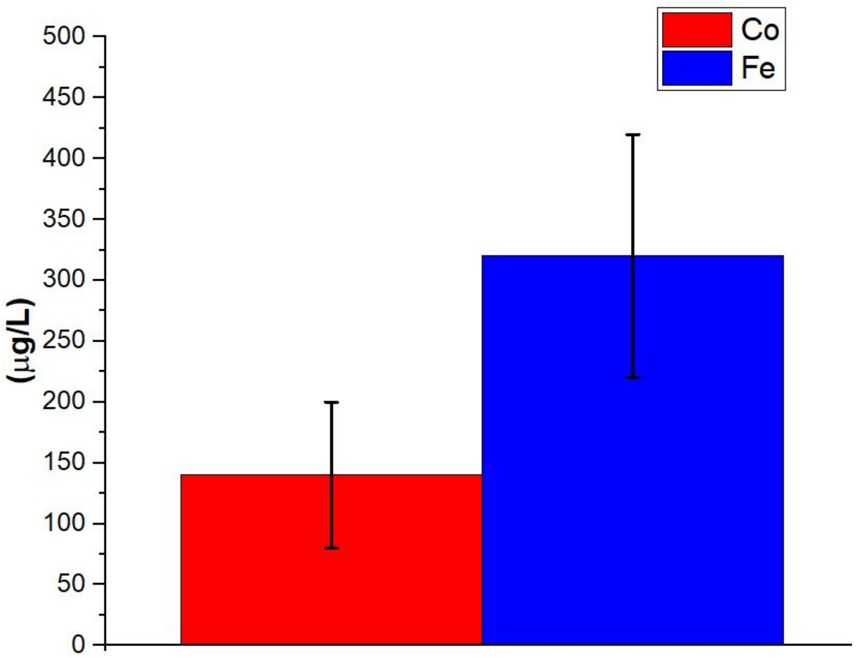

Figure S1. The total amount of Fe and Co ion release in simulated solution during 48h incubation.

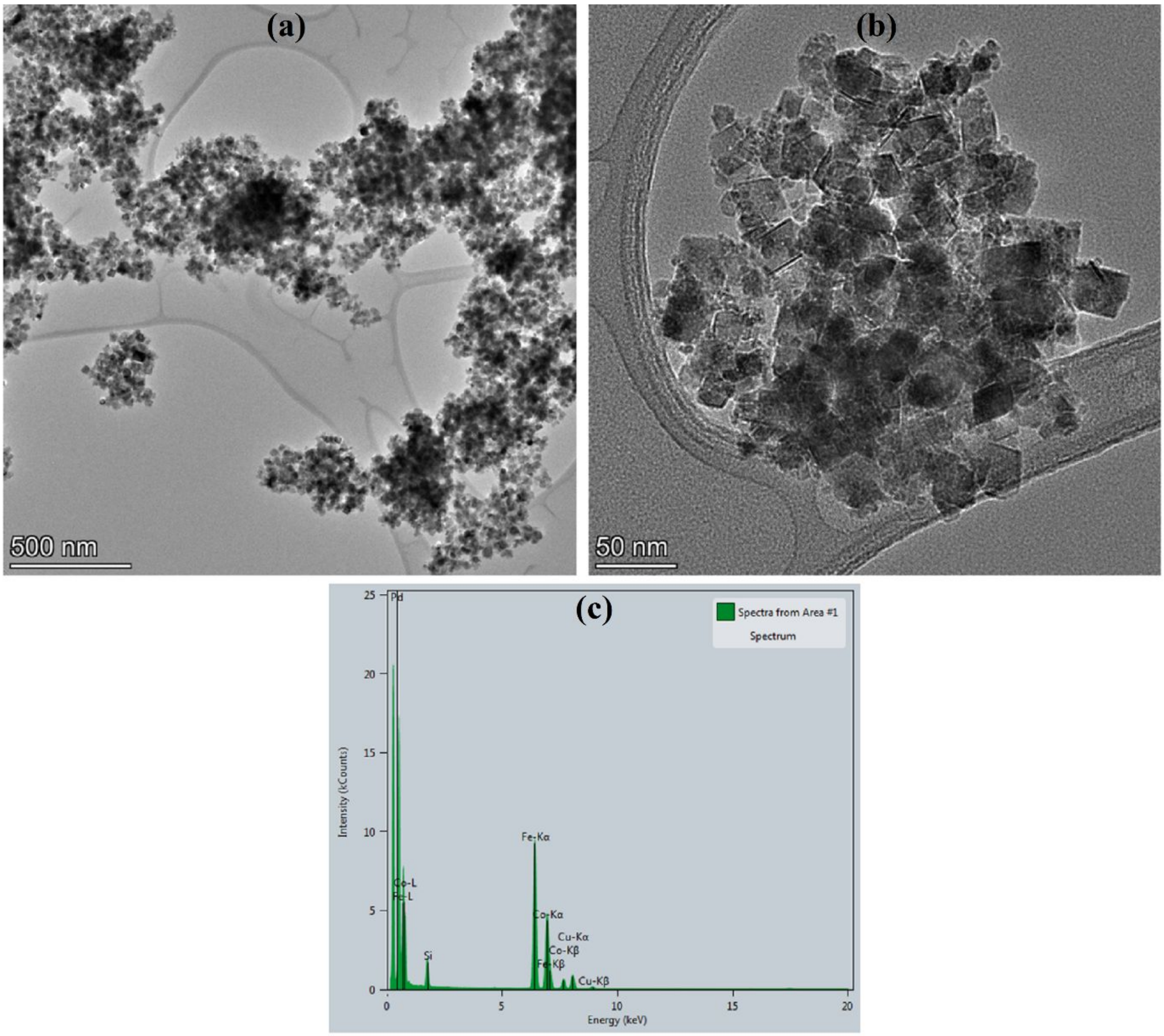

Figure S2. (a, b) TEM images of CFO-distributed nanoparticles at two different magnifications, (c) EDS elemental analysis of CFO-distributed nanoparticles from b.

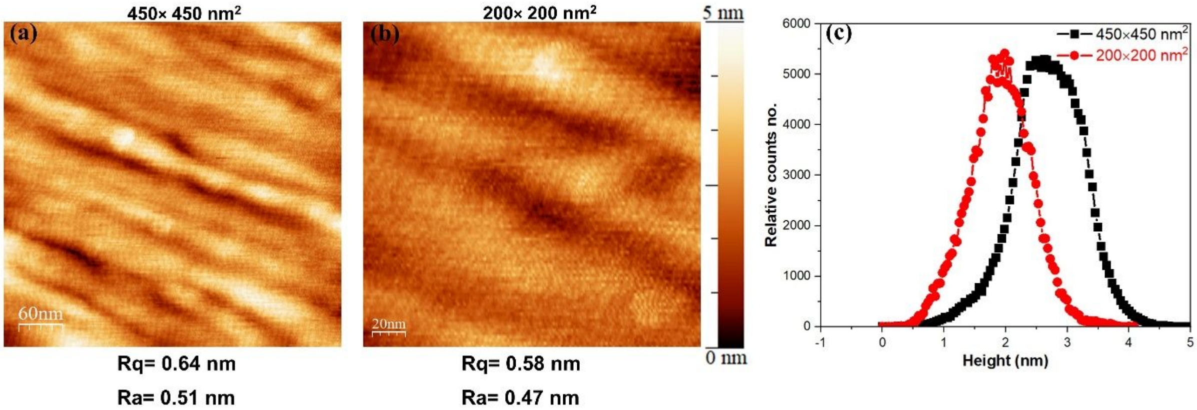

Figure S3. AFM topography images of CoCrMo mirror-like surface at two different magnifications (a) 450×450 nm^2 and (b) 200×200 nm^2, (c) corresponding histogram of topography distribution from a and b.

Sample preparation for current-potential measurement

20 mg of CFO nanoparticles were gently mixed with 150 µL of ethanol and 20 µL of Nafion (Nafion® 117, Sigma–Aldrich) [2, 3]. Then, the suspension was uniformly coated on 2×2cm^2 indium thin oxide (ITO,18-20 ohms/sq, techinstro) coated glass using a spin coater (Ossila, UK) with spin speed 800 rpm and spin time 20 seconds. As a final point, the samples were dried at 60 Cº for 1 hour. Based on this procedure, the most of top layers or nanoparticles surfaces are composed of the lowest Nafion compound, while the bottom coated layers of nanoparticles are mainly fixed in the Nafion mixture, as shown in **Figure S4b**. The current-potential (I-E) measurements were performed at 25Cº with a potentiostat instrument (AUTOLAB PGSTAT 30) with Ag/AgCl/KCl$_{3M}$ reference electrode (+222 mV vs. SHE) and a platinum wire as a counter electrode. The surface area of all exposed samples was carefully controlled to 0.5 cm^{-2}. All electrochemical measurements were accomplished after 1-hour immersion for the stabilization of the open circuit potential (OCP). The I-E measurements were carried out at a scan rate of 1 mV.s^{-1} at anodic potentials to reveal the oxidation or anodic reaction on the oxide nanoparticle surfaces.

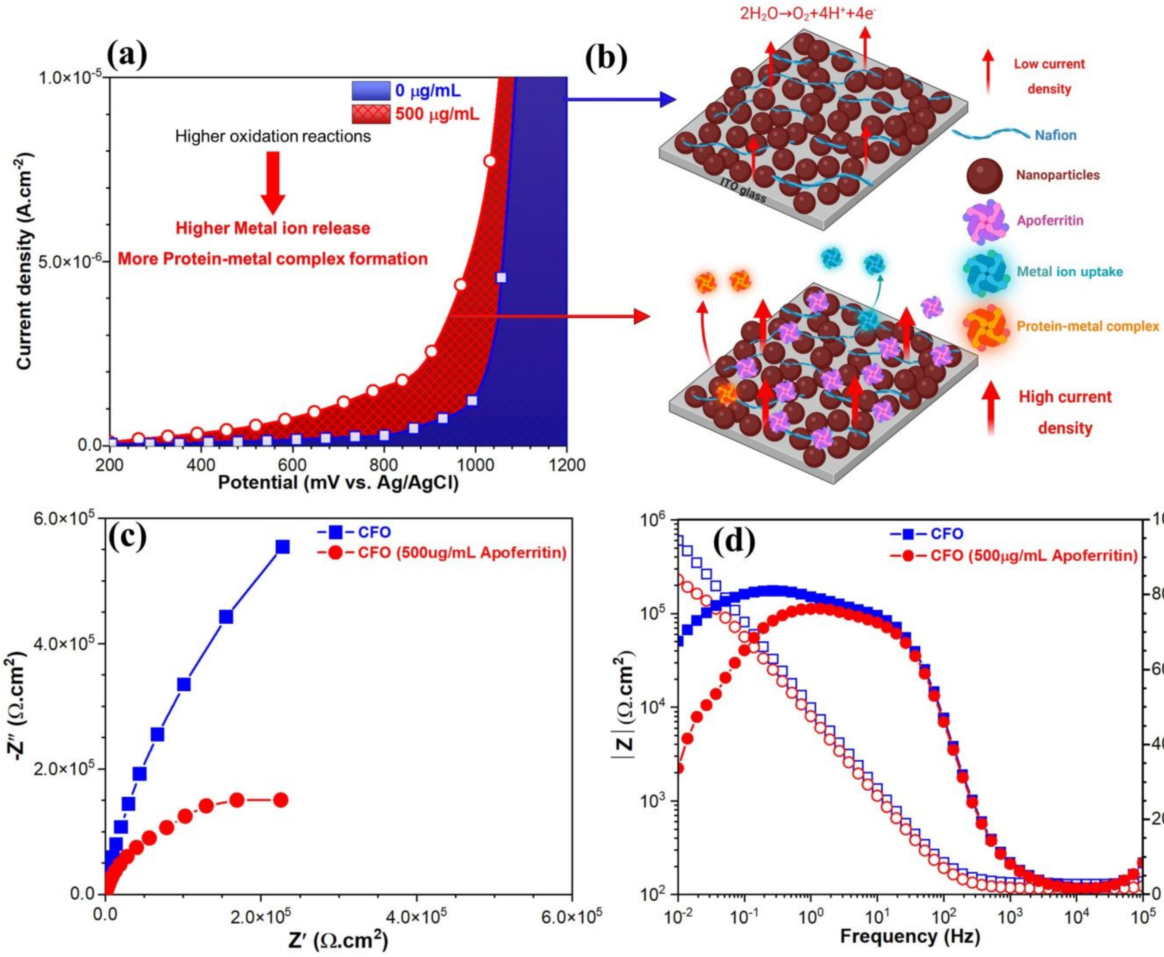

Figure S4. (a) I-V curves (anodic potential) of CFO nanoparticles after 1-hour immersion in 0.9% NaCl solution with and without the addition of apoferritin protein (500µg/mL) at 25 °C, (b) a schematic presentation of CFO nanoparticles+ Nafion® distribution on ITO glass which coated by drop-coating method (spin coater) and impact of protein molecules on enhancing the current density and then accelerating the biodegradation process, (c) Nyquist and (d) Bode phase/magnitude after 1-hour immersion in 0.9% NaCl solution with and without the addition of apoferritin protein (500µg/mL) at 25 °C.

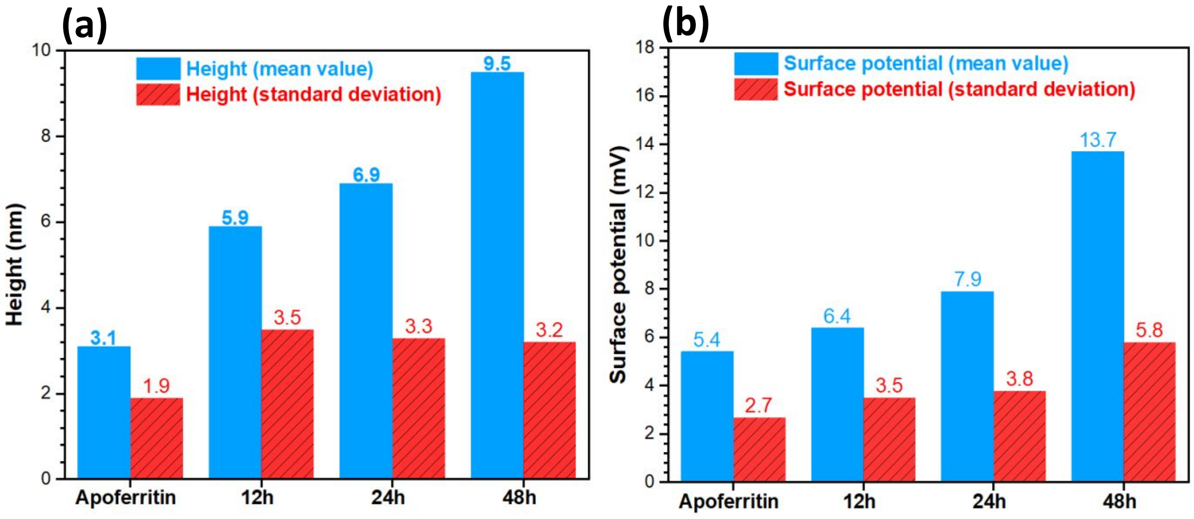

Figure S5. Mean value and standard deviation of (a) height and (b) electrical surface potential histograms in Figure 3i and 3j.

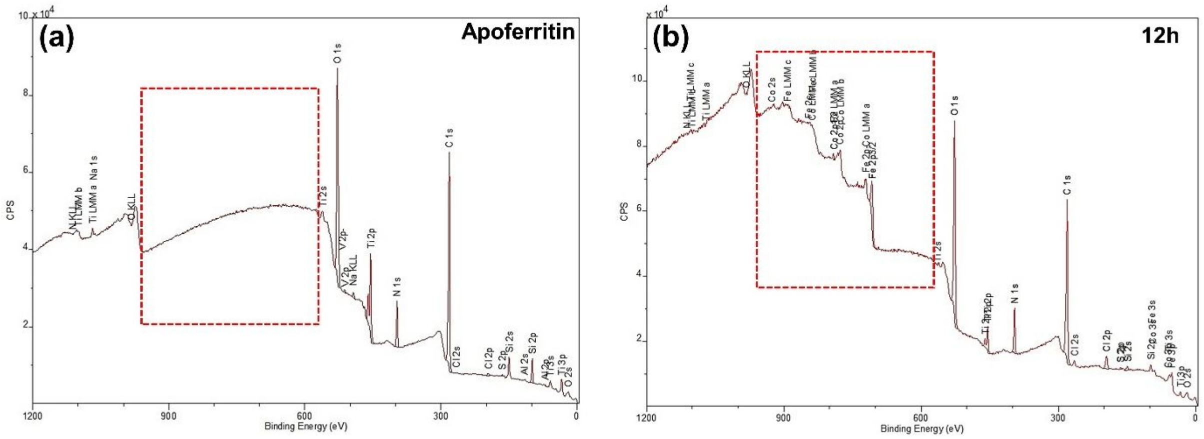

Figure S6. XPS survey spectra of adsorbed (a) fresh apoferritin (non-filled) and (b) filled-apoferritin at an incubated time of 12h. The red rectangle represents the absence of the Fe and Co signal in fresh adsorbed apoferritin, although the Fe and Co binding energy/signals are detected after a incubation time of 12h.

Table S1. Binding energy of Fe 2p in adsorbed filled-apoferritin monolayers at different incubation times.

Sample	Peak1 (eV)	Peak2 (eV)	Peak3 (eV)	Peak4 (eV)	Peak5 (eV)	Peak6 (eV)
**12h, 24h, 48h**						
γ-Fe_2O_3	709.85±0.25	710.89±0.15	711.92±0.3	713.10±0.17	714.29±0.23	-
Fe_3O_4	708.49±0.4	709.34±0.29	710.48±0.32	711.44±0.29	712.56±0.32	713.62±0.37
FeOOH	710.46±0.29	711.46±0.22	712.38±0.26	713.47±0.22	714.67±0.34	-

Table S2. Binding energy of Co 2p in adsorbed filled-apoferritin monolayers at different incubation times

**Sample**	**Co_3O_4 (eV)**	**CoO, CoOOH (eV)**
12h	779.54	780.72
24h	779.71	781.32
48h	779.56	780.76

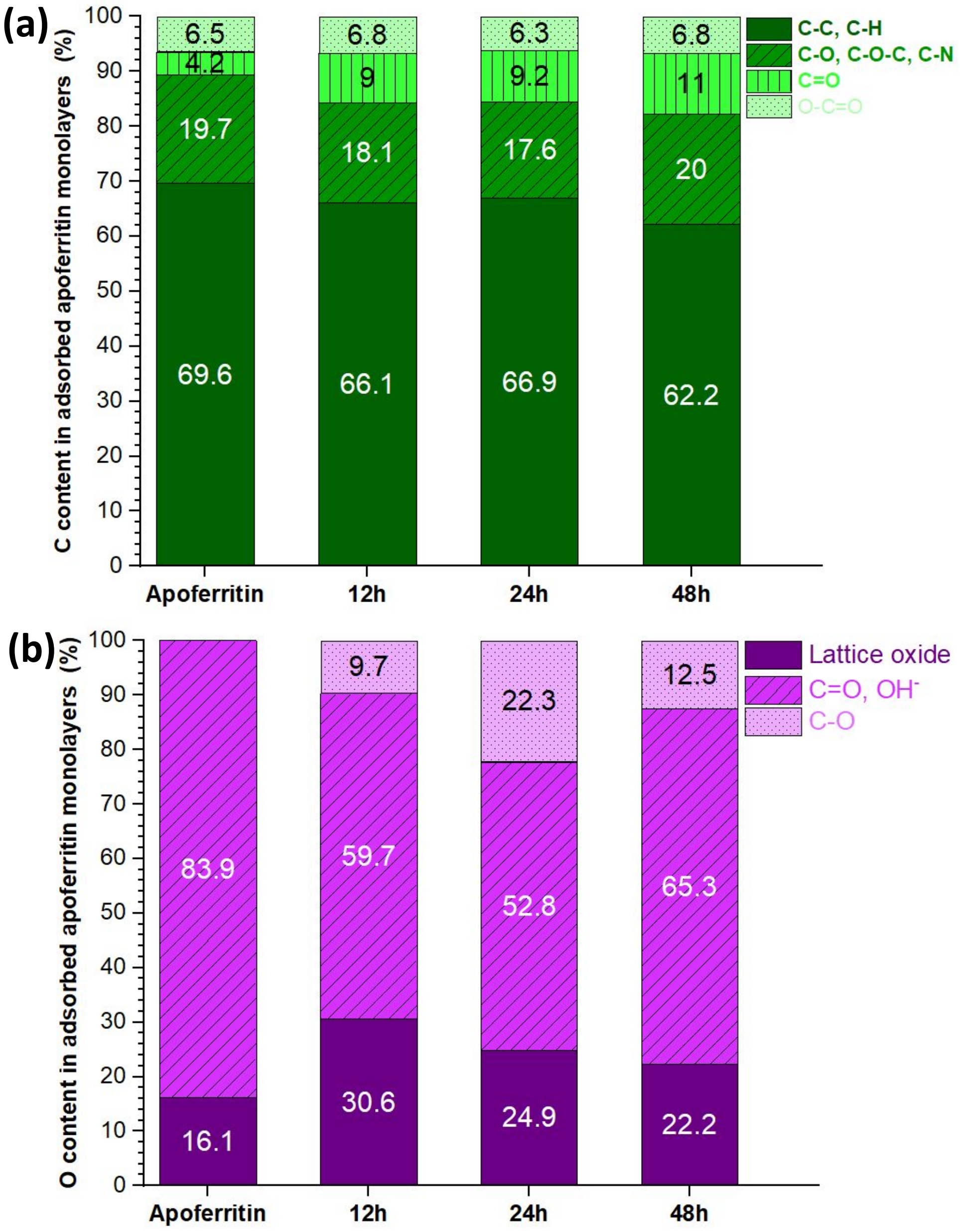

Figure S7. Amount of (a) C and (b) O in adsorbed apoferritin monolayers at various incubation times.

References

[1] S. Karimi, T. Nickchi, A.M. Alfantazi, Long-term corrosion investigation of AISI 316L, Co–28Cr–6Mo, and Ti–6Al–4V alloys in simulated body solutions, Applied Surface Science 258(16) (2012) 6087-6096.

[2] E. Rahimi, R. Offoiach, S. Deng, X. Chen, S. Pané, L. Fedrizzi, M. Lekka, Corrosion mechanisms of magnetic microrobotic platforms in protein media, Applied Materials Today 24 (2021) 101135.

[3] X. Zhao, Y. Fu, J. Wang, Y. Xu, J.-H. Tian, R. Yang, Ni-doped CoFe2O4 hollow nanospheres as efficient Bi-functional catalysts, Electrochimica Acta 201 (2016) 172-178.
